# Criterion-Related Validation of a Music-Based Attention Assessment for Individuals with Traumatic Brain Injury

**DOI:** 10.3390/ijerph192316285

**Published:** 2022-12-05

**Authors:** Eunju Jeong, Susan J. Ireland

**Affiliations:** 1 Department of Music Therapy, Graduate School, Ewha Womans University, Seoul 03760, Republic of Korea; 2 Uhealth Jackson Rehabilitation Care, Miami, FL 33136, USA

**Keywords:** melodic contour identification, attention assessment, criterion-related validation, traumatic brain injury, neurocognitive evaluation, neurorehabilitation

## Abstract

The music-based attention assessment (MAA) is a melody contour identification task that evaluates different types of attention. Previous studies have examined the psychometric and physiological validity of the MAA across various age groups in clinical and typical populations. The purpose of this study was to confirm the MAA’s criterion validity in individuals with traumatic brain injury (TBI) and to correlate this with standardized neuropsychological measurements. The MAA and various neurocognitive tests (i.e., the Wechsler adult intelligence scale DST, Delis–Kaplan executive functioning scale color-word interference test, and Conner’s continuous performance test) were administered to 38 patients within two weeks prior to or post to the MAA administration. Significant correlations between MAA and neurocognitive batteries were found, indicating the potential of MAA as a valid measure of different types of attention deficits. An additional multiple regression analysis revealed that MAA was a significant factor in predicting attention ability.

## 1. Introduction

Traumatic brain injury (TBI) is defined as damage to brain tissue caused by an external mechanical force and is characterized by loss of consciousness, post-traumatic amnesia, or objective neurological findings that are attributable to TBI upon a physical or mental examination [1]. Such damage may lead to permanent or temporary cognitive, socio-emotional, and/or sensory-motor impairment [2,3,4,5,6,7]; however, attention deficit has been reported to be the most common [8,9,10,11]. Impaired attention types vary from sustained attention [12,13,14,15] to selective attention [14,16,17,18] and more complex information processing, such as supervisory attention [18,19,20,21,22,23,24]. From a neuropathological perspective, primary and secondary head injuries (e.g., contusions in the sensory and frontal cortices, widespread axonal disruption) cause sensory disturbances and increased distractibility [2,25,26,27,28]. These disturbances result in attenuated and inaccurate attention performance [6,29,30,31]. Auditory information processing is considerably challenging [32,33,34,35,36,37] and attention performance rapidly decreases in the presence of auditory distraction [12,18,33,37,38,39].

Owing to this diverse range of attention deficits and their changes during the recovery process, a tailored approach to rehabilitation is necessary. However, the majority of existing attention batteries focus on attention in the visual modality, while a few measures utilize arithmetic information presented verbally, such as the WAIS-IV DST [40] and paced auditory serial addition test [41]. More importantly, attention involving the processing of incoming multi-layered auditory stimuli has not been well evaluated because the measures employ a single auditory stream which does not simulate the complexity of distracting sounds in auditory environments. Current attention batteries also lack assessment tasks that are hierarchically organized through the course of information processing, leading to inefficiency when multiple tests are administered. Therefore, attention measures for individuals with TBI need to embrace assessment tasks comprising various types and levels of cognitive demand embedded in a simulated realistic auditory environment.

Music-based attention assessment (MAA) is a melodic contour identification task, of which stimuli include pitches and rhythms. Both musical components are known to activate and utilize the cognitive as well as motor systems of patients with neurological disorders [18,42,43]. Specifically, melody perception requires involuntary and voluntary involvement of attention. Snyder and Alain [44] found that attention occurs involuntarily to process psychoacoustic features of sounds, and then voluntary attention follows to yield auditory stream segregation. Behavioral responses to music revealed the involvement of attention in music processing [45,46,47,48,49,50]. Additionally, another group of studies claimed that depending on the manner of listening to music (i.e., melody), specific brain regions modulating different types of attention are activated [51,52,53]. The brain regions are (a) the reticular formation, cerebellum, and thalamus (sub-cortical areas), primary sensory areas, motor areas, anterior cingulate and the prefrontal areas (cortical areas) for sustained attention, (b) the polysensory association areas (i.e., the junction of the occipital, parietal, and temporal regions) for selective attention, and (c) the anterior cingulate cortex, the parietal sulcus, and most of the frontal cortex for divided attention. Such findings together suggest that behavioral responses to music have a potential for measuring attentional ability and, thus, the appropriate use of music can be an effective measure of different types of attention ability [52,54,55,56,57,58,59].

The MAA was developed based on the neuroanatomical commonality between music perception and attention and aimed to measure different type and levels of attention involving auditory information processing in environmental contexts. The MAA was piloted with a small group of patients diagnosed with TBI (*n* = 15), reporting a wide range of item difficulty and good reliability [60]. The authors then revised the MAA to vary item difficulty and reflect a wider range of the continuum of attention ability existing in typical and clinical populations. Jeong [61] administered a revised 54-item version of the MAA to a larger group of young adults (*n* = 165) and patients with TBI (*n* = 22). Statistical validation showed promising construct validity and reliability, suggesting the elimination of nine problematic items and the partial reconstruction of the measure. In addition, comparisons of neurophysiological responses using functional near-infrared spectroscopy (fNIRS) were investigated in different age groups in typical [62,63] and clinical populations, such as patients with acquired brain injury and clinical liver disease [64,65]. This study investigated the criterion validity of the MAA as correlated with current neuropsychological attention measurements, that is, the Wechsler adult intelligence scale (WAIS-IV) digit span test, Conner’s continuous performance test, and the Delis–Kaplan executive functioning scale (D-KEFS) color-word interference test. The study also explored the potential of using the MAA as a predictor of cognitive capacity in neurorehabilitation.

## 2. Methods

### 2.1. Participants

Thirty-eight patients with TBI were voluntarily recruited from the health system of a hospital in South Florida according to convenience sampling. Participation was available to individuals diagnosed with traumatic brain injury and who scored less than 13 on the Glasgow coma scale (GCS) or, alternatively, a Rancho Los Amigos scale (RLAS) score greater than V at the time of brain injury assessment. Eligibility was also limited to patients who were non-professional musicians and had no visual or hearing impairment. The average age of the participants was 35.45 (*SD* = 14.79), and there was an average of 22.42 (*SD* = 33. 28) months since the date of brain injury. The participants’ levels of awareness as measured by GCS and cognitive functioning as measured by RLAS was an average of 5.78 (*SD* = 3.96) and 6.79 (*SD* = 2.44), respectively. The participants’ average years of education was 14.15 (*SD* = 3.77), with an average of 2.09 years of music education (*SD* = 2.31). Table 1 presents additional demographic characteristics.

### 2.2. Measures

Music-based attention assessment (MAA). MAA is a melodic contour identification task consisting of sustained, selective, and divided attention subtests. The melodic contours used to generate the test items included a series of five tones moving in one of three directions: ascending, stationary, or descending. One to three series of five tones were arrayed to form a single auditory stream, of which the presentation time ranged from 3–10 s. In the sustained attention-basic subtest, a set of five tones was presented as target contour. The number of tones in the target contour increases to include two to three sets of five tones in the sustained attention-advanced subtest. In the selective attention subtest, one to three sets of fives tones were presented with distraction (i.e., environmental sounds). In the divided attention subtest, two streams of one to two sets of five tones were presented concurrently, with one serving as the target contour and the other serving as a target-like distractor. Participants were asked to identify the target contour only in the divided attention-basic, while they were asked to identify both contours in the divided attention-advanced. In the present study, a 45-item MAA was utilized after eliminating 9 test items that showed statistical inconsistency [61]. The sound items of the MAA were produced using a music synthesizer (Korg N5EX, Korg Inc., Tokyo, Japan) and were programmed into a media player (iTunes version 10. Apple Inc., Cupertino, CA, USA) using a laptop computer amplified with speakers. Table 2 shows the number of test items, stimuli, context characteristics, and the given tasks.

Digit Span Test (DST). The DST, a subtest of the working memory index of the Wechsler Adults Intelligence Scale—Fourth Edition (WAIS-IV) [40], is one of the frequently used attention assessment in a neurorehabilitation setting [66,67]. The DST consists of digit span forward (DSF), digit span backward (DSB), and newly added digit span sequencing (DSS) tests. In the DST, participants recalled a spoken sequence of numbers: (1) in the same order that the examiner presented (DSF), (2) in inverse order (DSB), and (3) in ascending order (DSS). Each item in the DSF, DSB, and DSS consisted of two trials. If the participant responded correctly, one point was assigned for the success of each trial. A maximum score of 16 was obtained, with a maximum summed score of 48. The completion time of the DST was approximately ten minutes.

Color-Word Interference Test (CWIT). The CWIT is one of the nine subtests of the Delis–Kaplan executive function system (D-KEFS) [68], which is often used in clinical settings to assess mild brain damage in general and mild frontal lobe involvement in particular [69]. The CWIT comprises four conditions: naming, word reading, inhibition, and inhibition/switching. It assesses the ability to inhibit an overlearned verbal response using colors and letters (i.e., names of colors printed in a congruent or incongruent color ink). The total number of uncorrected errors, corrected errors, and time for completion was recorded. The scaled score for the completion time was used in this study. The time required to complete the CWIT was 5–10 min.

Conner’s Continuous Performance Test. The Conner’s continuous performance test (CPT) is widely used to measure various aspects of attentional behavior, such as inattentiveness, impulsivity, activation, and vigilance. The given task is to press a space bar or click when letters appear on the computer screen, excluding the letter “X”. For this study, eight measures that are sensitive to explain the response patterns of inattentiveness were used, including omissions, commissions, hit reaction time, hit reaction time standardized error, variability, detectability, hit reaction time inter-stimulus-interval change, and hit standardized error inter-stimulus-interval change. The CPT consisted of six blocks and three sub-blocks, each containing 20 letters [70], and the completion time was approximately 14 min.

### 2.3. Procedures

The Institutional Review Board (IRB) of the University of Miami approved this study (no. 20081058). Once permission was obtained from the patient recruitment site, a neurorehabilitation programme director was identified as eligible to participate in the study. The researcher and staff in the neurorehabilitation programme met with the patients and/or caregivers and explained the purpose of the study. If the patients and/or caregivers showed an interest in participating, the programme staff arranged a time and place for the experiments. At the time of administration, the participants completed a demographic questionnaire and completed the MAA. The MAA started with a practice session in which participants were asked to identify the directions of the contours until they could correctly identify more than 80% of the directions. In a test session, each of the five subtests was initiated with a brief instruction in terms of the task characteristics given in each subtest and how to respond to test items. Participants were also instructed to identify the directions of the target contours by circling the arrow corresponding to the contour direction given in the answer sheet. In the sustained attention-basic and -advanced, selective attention, and divided attention-basic, participants were asked to identify the direction of a target contour, while they were asked to identify the directions of two target contours. A total amount of time to complete the MAA was approximately 30 min. The volume was the same for each participant, and the researcher was able to provide guidance and answer all questions. The experiment was performed in a sound-proof room to control for other noises. The ambient light and temperature remained constant throughout the experimental sessions. Within two weeks prior to or after administration of the MAA, the staff in the neurorehabilitation programme conducted a psychological evaluation with the patients using the WASI-IV DST, D-KEFS CWIT, and Conners’ CPT.

### 2.4. Statistical Analysis

This study employed a descriptive-correlational design to examine the criterion validity between the MAA and different types of cognitive function. Data from the score of MAA and cognitive measures were subjected to correlation analysis. Owing to the patients’ availability for psychological evaluation or cancellation, an unequal number of participants were administered the experimental measures, where 22 patients underwent CCPT and 38 patients took the WAIS-IV DST and D-KEFS CWIT. In addition, a regression analysis was performed to examine whether the MAA predicted cognitive capacity using demographic variables.

## 3. Results

### 3.1. Correlation Analysis

A total of 38 participants underwent the MAA and a set of neurocognitive assessments. Table 3 presents the descriptive statistics of the measurements.

A correlation analysis was performed between the MAA and attention batteries. The variables taken from each of the four measurements included: (1) the five subtests of the MAA, reconstructed by Jeong (2013); (2) scaled scores of the three subtests and total score of the WAIS-IV DST; (3) scaled scores of the four conditions of the D-KEFS CWIT; and (4) T scores of eight inattention measures of the CCPT (compared with the typical norm). The results showed significant correlations between the tests (Table 4).

Each of the three subtests of the DST was significantly correlated with the four subtests of the MAA, except for sustained attention-basic. These findings indicate the potential of the MAA in measuring attention and working memory capacity; however, the type and level of attention that the researchers obtained were not as sensitively detected by the DST, as expected. The task characteristics given in the DST and MAA were different in terms of information processing strategy, yielding a significant correlation, but were not task-specific. The sustained attention-basic subtest was an exception, possibly because of the low information load compared to each of the three DSTs.

The three conditions of the CWIT, color naming, word reading, and inhibition, were significantly correlated with sustained attention-advanced, selective attention, divided attention-basic, and divided attention-advanced on the MAA. As shown by the DST, sustained attention-basics were not significantly correlated. There was a tendency for the *p*-values to become more significant between the MAA and cognitive tests requiring a lower or higher level of cognitive demand. For example, color naming and word reading conditions were more highly correlated with sustained attention-advanced and selective attention than between inhibition and inhibition/switching conditions and divided attention-basic. The divided attention-advanced of the MAA was correlated only with the inhibition/switching condition, indicating shared task characteristics and high cognitive demand between the two subtests. The inhibition condition correlated with the overall subtests of the MAA, indicating shared task characteristics requiring a focus on the target over irrelevant stimuli.

The eight inattention measures of the CCPT were subjected to a correlation analysis and yielded a significant correlation between hit RT SE, variability, hit RT ISI change, and hit SE ISI change. The selective attention and divided attention-advanced subtests correlated with hit RT SE and variability, indicating that the two music subtests were more sensitive to measurements of response speed consistency and variability over the course of the assessment. The divided attention-basic subtest correlated with the hit RT ISI change and hit SE ISI change, indicating changes in reaction time and its consistency over time. Overall, a higher level of subtests of the MAA, in terms of attention hierarchy, was significantly related to the inattention measures of the CCPT, indicating the potential of the MAA as an indicator of auditory inattention.

### 3.2. Multiple Regression Analysis

A multiple regression (MR) analysis was conducted to determine whether the MAA performance and demographic variables predicted the total value of cognitive capacity. The MAA performance was calculated by summing the four subtest scores, and cognitive capacity was scaled to the total scores of the WAIS-IV DST. Prior to the MR analysis, a correlation analysis was performed. Table 5 presents the correlations among the demographic variables, MAA performance, and cognitive capacity.

The findings showed that the MAA performance and years of education explained a significant amount of the variance in the cognitive capacity as measured by the WAIS-IV DST (*F* (2, 34) = 6.31, *p* < 0.01, *R*^2^ = 0.52, *R*^2^*_adjusted_ =* 0.23). The analysis showed that MAA performance significantly predicted general cognitive capacity (*β* = 0.39, *t* (34) = 2.46, *p* < 0.05), whereas years of education did not (*β* = 0.23, *t* (34) = 1.47, *p* = 0.15). The years of music education were subjected to an MR analysis because there was a significant correlation between education and music education. The change in R2 was not significant (*F* (3, 33) = 0.02, *p* = 0.35), indicating that the addition of this variable did not increase the total variance in cognitive capacity. Table 6 shows the MAA performance with the other variables, accounting for the variance in general cognitive capacity.

## 4. Discussion

This study investigated the criterion validity of the MAA and existing neurocognitive batteries. The results showed that melody identification tasks with different textures, which mimic a real auditory environment, can measure attention ability in general. Significant correlations with several subtests of neuropsychological measures explained the criterion validity of the MAA. A significant correlation between the CWIT inhibition/switching condition and the MAA divided attention-advanced subtest indicated that the MAA may have the potential to differentiate which type and level of attention is affected in individuals with TBI. In addition, the hit RT SE and variability measure of the CCPT were significantly correlated only with the MAA selective attention and divided attention subtests. This finding indicates that dysfunctions in inattentiveness and variability that contribute to increased distractibility were possibly detected by the MAA. Additionally, the MR analysis demonstrated the potential of using MAA as a predictor of cognitive capacity.

### 4.1. MAA as a Measure of Attention and Working Memory

Significant correlations with the WAIS-IV DST suggest the potential of the MAA to measure attention and working memory capacity. Acoustic properties and structural elements of music in the MAA utilize bottom-up resources in the brain and activate regions of attention and working memory [52,71,72]. The given tasks designed for the purpose of subtests of the MAA increase the relevance of the stimuli and may attract more attention resources to the stimuli [73]. Stimulus- and goal-driven attention interact in a reciprocal manner [74,75], and the intercommunication between the two processes is key to providing a rationale for using the MAA as an attention assessment tool for TBI.

Despite evidence of the MAA being an appropriate assessment task, the type and level of attention were not sufficiently differentiated through the administration of the DST only. Considering that the DST utilizes a single auditory stream that is verbally presented, the task characteristics of the DST may yield significant correlations but may not be specific to the subtests addressing the assessment of different attention types. In addition, a single subtest (sustained attention-basic) of the five subtests of the MAA did not show a statistically significant correlation with the DST. This exception was possibly due to the relatively low cognitive load of the sustained attention-basic, suggesting the need for revision of the items in the subtest.

### 4.2. MAA as a Measure of Different Types of Attention

The color–word interference test is a measure of selective and alternating attention. It was significantly correlated with the four subtests of the MAA. The coefficient values tended to increase as the hierarchy increased in attention types. Tasks with a relatively low cognitive load, such as the sustained attention-advanced and word reading condition and the divided attention-basic and inhibition/switching condition, were more highly correlated than the other pairs. Importantly, the divided attention-advanced subtest was correlated solely with the inhibition/switching condition, which required the highest cognitive demand among the four conditions of the CWIT. These findings suggest that the tasks in each of the five subtests of the MAA are hierarchically organized to sensitively measure the level and type of attention.

Individuals utilize and activate different attention strategies while listening to various textures in which musical structures are arrayed. According to the music attention model [45], listening to two or more music streams simultaneously activates different attention types, such as alternating and divided attention. Neurological evidence also supports the view that multi-voice music listening can lead to the activation of neural regions that house each type of attention [51,52,72,76]. These findings support the aforementioned studies reporting the connection between music and attention, and further suggest its use as a different type of auditory attention. Specifically, the correlation with the CWIT articulated the role of the MAA in measuring different levels and types of attention, which was not shown in the relationship with the DST. The given tasks included various musical elements of the MAA.

### 4.3. MAA and the Distractibility Assessment

The eight inattention measures of the CCPT were subjected to a correlation analysis and yielded a significant correlation between hit RT SE, variability, hit RT ISI change, and hit SE ISI change. The selective attention and divided attention-advanced subtests were correlated with hit RT SE and variability, indicating that the two subtests of the MAA were more sensitive in measuring response speed consistency and its variability over the course of the assessment. The divided attention-basic subtest was correlated with hit RT ISI change and hit SE ISI changes, indicating changes in reaction time and its consistency over time. Overall, the higher level of the subtests of the MAA in terms of attention hierarchy was significantly related to the inattention measures of the CCPT, suggesting the potential of MAA as an indicator of auditory inattention.

### 4.4. The MAA as a Predictor of Cognitive Capacity

The MAA performance with years of education, rather than severity, age, or time since brain injury, was found to predict general cognitive capacity. Several factors are strongly associated with cognitive deficits and recovery following a TBI. Among various individual variables, brain injury severity is considered the main factor that determines cognitive performance out of various individual variables [77,78]. The significant correlation found between cognitive capacity and level of education was inconsistent with previous studies and may indicate that individuals with a higher level of education may utilize a more focused effort learned from their educational experience [79]. However, since the MAA utilizes novel materials and tasks rather than numbers, words, or geometrical figures with complex verbal instructions, it is difficult to interpret the influence of learned knowledge.

Interestingly, the years of music education were not found to account for the additional variance in existing cognitive capacity, although the MAA primarily utilized music-based materials. This finding may indicate that the processing of music-based information given in the MAA does not necessarily require a specific level of music sensitivity or talent and, thus, suggests the potential of using the MAA for people who do not have experience in musical training.

## 5. Conclusions

Evidently, the MAA utilizes melodic identification tasks with different musical textures and task demands to measure general cognitive capacity, different types of attention, as well as distractibility which is a causal factor of inattentiveness. Melodic contours weaved into different textures in the MAA may closely mimic a real-world auditory environment and thus provide an ecologically valid measure of auditory attention. Additional structural elements of music, such as instrumental timbres and pitch range, provide a method for randomizing and creating music assessment items and further extends them to the generalized item pool of attention training. One of the advantages of the MAA is that the receptive modality of music requires minimal prerequisite abilities and, therefore, may be applied to individuals who have either temporary or permanent difficulty in understanding complex verbal instructions. In addition, music processing enhances cognitive functions by affecting elevation, which helps maintain an optimized arousal state for assessment and prevents cognitive fatigue during the assessment process.

This study has several limitations, including its small sample size (*n* = 38) and the skewed female-to-male ratio. It seems to reflect the epidemiology of TBI, reporting that men are approximately 40% more likely to suffer a TBI compared with women [80,81,82], In this study, we found no sex-related differences in any of the musical or cognitive–behavioral responses. However, in future studies, it would be necessary to balance the gender ratio with an increase in sample size. Another limitation of the study could be a non-probability convenience sampling from a single medical center. Although it is often used in clinical research [83], care should be taken in attempting the generalization of data.

Future suggestions include reliability and validity investigations with a larger group of people in which the normal distribution of the recovery phase and injury severity is assumed and who are from various sociocultural backgrounds. A comparison of attention performance depending on the variability of neuropathology could be evaluated to validate the use of the MAA. Parallel observation using brain imaging techniques would also contribute to establishing clinical evidence and a model of music and attention. Lastly, cognitive performance is measured by externalized behaviors; therefore, a greater variety of behavioral responses related to attention function, such as reaction time and task completion time, must be included in the MAA. The development of a computerized version of the MAA would provide appropriate solutions for a multifaceted measurement of attention function.

Neurocognitive science has illuminated the cognitive abilities that underlie music-related activities. Music is believed to be a powerful tool for people with and without previous music training and talent, and therefore it is important to identify the basic units of behavior involving listening and making music, and investigating their relationship with cognitive behaviors. The development, validation, and frequent use of a tangible measure of music behavior could provide alternatives to prove the efficacy of music therapy in enhancing a broad spectrum of functional behaviors.

## Figures and Tables

**Table 1:** Demographic characteristics.

		*n*	%
Gender	Male	30	75
	Female	10	25
Ethnicity	Caucasian	5	12.5
	African American	12	30
	Hispanic	23	75
Data since brain injury	Less than a year	10	25
	1 to 2 years	12	30
	More than 2 years	18	45

**Table 2:** Structure of the 45-item MAA.

Subtests		Item#	Stimuli	Contexts	Tasks
Sustained Attention	Basic	4	A set of five tones	A single auditory stream	To identify the direction(s) of the melodic contour
	Advanced	13	Two to three sets of five tones		
Selective Attention		6	One to three set(s) of five tones and environmental sounds	Two auditory streams: One conveying task-relevant information and the other delivering distracting sounds	To identify the direction(s) of the melodic contour in the presence of distraction
Divided Attention	Basic	16	One to two set(s) of five tones	Two auditory streams: both conveying task-relevant information	To identify the direction(s) of the melodic contour in the presence of more competing sounds
	Advanced	6	Three sets of five tones		To identify two direction(s) of melodic contours

**Table 3:** Descriptive statistics of the MAA and neurocognitive batteries.

		M	SD	Minimum	Maximum
MAA	Sustained Attention-Basic	3.35	1.21	0	4
	Sustained Attention-Advanced	8.18	4.09	0	14
	Selective Attention	3.40	1.88	0	6
	Divided Attention-Basic	5.48	3.99	0	16
	Divided Attention-Advanced	0.08	0.27	0	1
	Total	20.48	9.76	2	41
DST	Forward	7.74	3.49	2	17
	Backward	7.87	2.30	3	15
	Sequencing	6.47	2.68	2	14
	Total	5.32	2.16	1	11
CWIT	Color naming	6.24	4.26	1	13
	Word reading	6.49	4.51	1	14
	Inhibition	7.08	4.94	1	15
	Inhibition/Switching	6.24	4.83	1	15
CCPT	Omissions	82.02	78.08	40.86	321.03
	Commissions	48.72	11.18	33.38	73.90
	Hit RT	56.05	16.75	16.79	86.44
	Hit RT SE	57.00	17.61	33.15	94.71
	Variability	55.62	14.67	30.11	89.78
	Detectability	47.60	11.16	21.43	66.40
	Hit RT ISI Change	51.01	12.47	24.48	90.18
	Hit SE ISI Change	50.17	9.65	35.92	72.54

Note: *n* = 38 (MAA), *n* = 38 (CWIT, DST), and *n* = 22 (CCPT); RT, reaction time; SE, standardized error; ISI, inter-stimulus interval.

**Table 4:** Correlations between the MAA and neurocognitive batteries.

	MAA	Sustained Attention-Basic	Sustained Attention- Advanced	Selective Attention	Divided Attention-Basic	Divided Attention- Advanced	Total
Subtests							
DST	Forward	0.17	0.31	0.39 *	0.36 *	0.39 *	0.40 *
	Backward	0.03	0.54 ***	0.52 **	0.41 *	0.45 **	0.51 **
	Sequencing	0.17	0.35 *	0.46 **	0.32 *	0.39 *	0.40 *
CWIT	Color naming	−0.00	0.50 **	0.50 **	0.41 *	0.15	0.48 **
	Word reading	0.01	0.51 **	0.47 **	0.39 *	0.15	0.47 **
	Inhibition	−0.15	0.46 **	0.43 **	0.43 **	0.24	0.44 **
	Inhibition/Switching	−0.07	0.36 *	0.36 *	0.44 **	0.38 *	0.40 *
CCPT	Hit RT SE	0.08	−0.36	−0.43 *	−0.20	−0.38 *	−0.39 *
	Variability	0.02	−0.44 *	−0.54 **	−0.34	−0.49 **	−0.48 **
	Detectability	0.01	0.17	0.25	−0.01	0.17	0.23
	Hit RT ISI Change	0.13	0.24	0.25	0.45 *	0.32	0.21
	Hit SE ISI Change	0.21	0.32	0.38 *	0.51 **	0.42 *	0.28

Note: *n* = 38 (CWIT, DST), *n* = 22 (CCPT); * *p* < 0.05, ** *p* < 0.01, *** *p* < 0.001.

**Table 5:** Correlations between the demographic variables, MAA, and neurocognitive batteries.

	Age	Date	Education	Music Education	MAA	DST
Age	-					
Date	0.13	-				
Education	0.16	0.14	-			
Music education	0.29	0.29	0.50 **	-		
MAA	−0.64	0.65	0.37 *	0.16	-	
DST	0.23	0.03	0.32 *	0.13	0.40 *	-

Note: *n* = 38 for this analysis, * *p* < 0.05, ** *p* < 0.01.

**Table 6:** Comparison of the MR models.

Model	Variables	*R* ^2^	*R*^2^ Change	*F*	*β*
1	MAA performance	0.47	0.23	10.13 ^**^	0.47 ^**^
2	MAA performance	0.52	0.05	6.31 ^**^	0.39 ^*^
	Education				0.23
3	MAA performance	0.54	0.02	4.50 ^**^	0.38 ^*^
	Education				0.31
	Music education				−0.16

Note: *n* = 38 for this analysis, * *p* < 0.05, ** *p* < 0.01.

## Data Availability

The data presented in this study are available upon request from the corresponding author.
